# Enhanced CO_2_ capture potential of UiO-66-NH_2_ synthesized by sonochemical method: experimental findings and performance evaluation

**DOI:** 10.1038/s41598-023-47221-6

**Published:** 2023-11-14

**Authors:** Amir Kazemi, Fatemeh Moghadaskhou, Mahyar Ashourzadeh Pordsari, Faranak Manteghi, Azadeh Tadjarodi, Ahad Ghaemi

**Affiliations:** 1 Research Laboratory of Inorganic Chemistry and Environment, Department of Chemistry, Iran University of Science and Technology, Tehran, 16846-13114 Iran; 2 Research Laboratory of Inorganic Materials Synthesis, Department of Chemistry, Iran University of Science and Technology, Tehran, 16846-13114 Iran; 3 School of Chemical, Petroleum and Gas Engineering, Iran University of Science and Technology, Tehran, 16846-13114 Iran

**Keywords:** Environmental chemistry, Chemical engineering

## Abstract

The excessive release of greenhouse gases, especially carbon dioxide (CO_2_) pollution, has resulted in significant environmental problems all over the world. CO_2_ capture technologies offer a very effective means of combating global warming, climate change, and promoting sustainable economic growth. In this work, UiO-66-NH_2_ was synthesized by the novel sonochemical method in only one hour. This material was characterized through PXRD, FT-IR, FE-SEM, EDX, BET, and TGA methods. The CO_2_ capture potential of the presented material was investigated through the analysis of gas isotherms under varying pressure conditions, encompassing both low and high-pressure regions. Remarkably, this adsorbent manifested a notable augmentation in CO_2_ adsorption capacity (3.2 mmol/g), achieving an approximate enhancement of 0.9 mmol/g, when compared to conventional solvothermal techniques (2.3 mmol/g) at 25 °C and 1 bar. To accurately represent the experimental findings, three isotherm, and kinetic models were used to fit the experimental data in which the Langmuir model and the Elovich model exhibited the best fit with R^2^ values of 0.999 and 0.981, respectively. Isosteric heat evaluation showed values higher than 80 kJ/mol which indicates chemisorption between the adsorbent surface and the adsorbate. Furthermore, the selectivity of the adsorbent was examined using the Ideal Adsorbed Solution Theory (IAST), which showed a high value of 202 towards CO_2_ adsorption under simulated flue gas conditions. To evaluate the durability and performance of the material over consecutive adsorption–desorption processes, cyclic tests were conducted. Interestingly, these tests demonstrated only 0.6 mmol/g capacity decrease for sonochemical UiO-66-NH_2_ throughout 8 consecutive cycles.

## Introduction

Anthropogenic CO_2_ emissions are closely linked to society's energy consumption and how it is provided. Global warming is primarily caused by the widespread emission of greenhouse gases and the high concentration of CO_2_ in the atmosphere caused by the use of fossil fuels^1^. Emissions of greenhouse gases have increased significantly in recent years^2–4^. Climate scientists have warned that greenhouse gas emissions are at an all-time high, resulting in unprecedented levels of global warming^5–8^. Since pre-industrial times, the global average temperature has increased by roughly 1.1 °C. The global temperature is projected to rise by 1.5 degrees Celsius by 2050 over pre-industrial levels^9,10^. In the coming decades, climate change may lead to unprecedented natural disasters on Earth. Global efforts have been made to reduce atmospheric CO_2_ concentrations to meet this challenge^11–13^. Due to an increased awareness of the link between atmospheric CO_2_ concentrations and global warming, research activities related to CO_2_ capture, storage, and utilization (CSU) have increased worldwide^14,15^. The most common methods for absorbing CO_2_ are chemical absorption by amine compounds, physical absorption by solid adsorbents, and membrane separation by diffusion^16–18^. Each of these methods has its advantages and disadvantages. Despite its simplicity and wide application, chemical absorption using amine compounds has several disadvantages, including high solvent consumption, a high energy requirement, and corrosion problems^19–21^. In contrast, membrane separation can significantly reduce energy consumption for CO_2_ absorption, but its high cost poses serious limitations^22,23^. Despite this, it appears that solid compounds are the most promising method for absorbing CO_2_^24,25^. In order to achieve this goal, activated carbon^26,27^, carbon molecular sieves (CMS)^28,29^, polymers, aluminophosphates (AlPOs)^30,31^, aluminosilicate zeolites^32,33^, covalent-organic frameworks (COFs)^34,35^, and recently, an entirely new class of porous materials, metal–organic frameworks (MOFs)^36–38^ have been widely considered as alternative technologies.

MOFs are porous materials comprising metal-inorganic nodes, called secondary building units (SBUs), and connected by rigid organic linkers. In the past two decades, research in MOFs has significantly broadened the range of applications for these porous and crystalline materials due to their exceptional storage, separation, and catalytic properties^39–43^. Having a unique and tunable structure, with precise control over pore size, shape, and functionality, these materials are highly promising for a variety of applications. The high surface area of MOFs allows for a high degree of gas and molecule adsorption. As a result of this property, MOFs are particularly suitable for gas storage, separation, and purification, as well as for catalysis, drug delivery, and sensing applications^44–47^. Research is currently focused on the development of new MOFs with improved properties, the exploration of their potential applications, and the advancement of their practical implementation in real-life environments. Different methods can be used to synthesize MOFs, including solvothermal, hydrothermal, microwave, mechanochemical, sonochemical, and electrochemical synthesis^48–52^. The choice of the method will depend on the specific properties of the MOF to be synthesized and its intended application. Each of these methods has its advantages and disadvantages. A powerful technique for synthesizing MOFs is sonochemical synthesis^53,54^. In this method, high-frequency ultrasonic waves are used to induce cavitation and tiny bubbles in the solution containing the metal and organic precursors. A high temperature and pressure are generated when these bubbles collapse, resulting in the formation of MOF crystals. In comparison with other conventional methods, sonochemical synthesis can produce MOFs in a relatively short period. Ultrasonic waves can accelerate the nucleation and growth of MOFs, resulting in a faster synthesis process. Furthermore, sonochemical synthesis can produce MOFs with a narrow size distribution, which is critical for a variety of applications. In addition to its high purity and crystallinity, sonochemical synthesis can produce MOFs of high purity. Using ultrasonic waves to generate high-energy conditions can facilitate the formation of well-defined MOF structures with minimal impurities. As a whole, sonochemical synthesis is a promising method for rapid, efficient, and high-quality synthesis of MOFs, making it a valuable tool for the development of new MOFs and their applications^55–58^.

In this study, a new sonochemical synthesis of UiO-66-NH_2_ has been performed using ZrCl_4_, 2-aminoterephthalic acid, and N, N-dimethylformamide, and the effect of particle morphology and size on gas adsorption of CO_2_ has been examined. Additionally, isothermic heat analysis and evaluation of different isotherm models have been performed to evaluate the adsorption performance. Using the Ideal Adsorbed Solution Theory (IAST), the selectivity of the sonochemically synthesized UiO-66-NH_2_ under conditions mimicking flue gas compositions has been quantified. Last but not least, cyclic performance experiments were conducted over a series of 8 repeated cycles to verify the durability and consistency of the adsorbent.

## Experimental section

### Material

Commercially available reagent grade chemicals were used without further purification. Zirconium (IV) chloride (ZrCl_4_, ≥ 99.5%), 2-amino terephthalic acid (NH_2_-BDC, ≥ 99.0%), and N, N-dimethylformamide (DMF) were obtained from Sigma Aldrich Co. Ltd. Hydrochloric acid (HCl, 37%), and absolute methanol (MeOH) were purchased by Merck Company. Deionized water (> 18 MΩ cm) was used throughout the whole experiment.

### Material characterization

FT-IR spectra were obtained from Shimadzu S 8400 infrared spectrophotometers using KBr pellets with a resolution of 4 cm^−1^ between 4000 and 400 cm^−1^. An ultrasonic generator (Elma-Hans Schmidbauer GmbH & Co.KG; D-78224 Singen, Germany), operating at 60 Hz with a maximum power output of 550 W, was used for the ultrasonic irradiation. FE-SEM, and EDX elemental mapping were conducted using the Zeiss Sigma 300 equipment. TEM was carried out by Philips EM 208S. Powder X-ray diffraction (PXRD) perusals were accomplished by STOE-STADV X-ray diffractometer with copper irradiance (Cu Kα, λ = 0.154 nm emission, 40 kV/40 mA current, and 3°/min scanning rate). N_2_ adsorption/desorption isotherms of the synthesized samples were investigated by the BET technique (Microtrac Bel Corp Belsorp mini II). Thermal gravimetric analysis (TGA) was conducted by STA 504 equipment from room temperature to 700 °C in air with a heating rate of 5 °C min^−1^.

### Material preparation

**Scheme 1:**
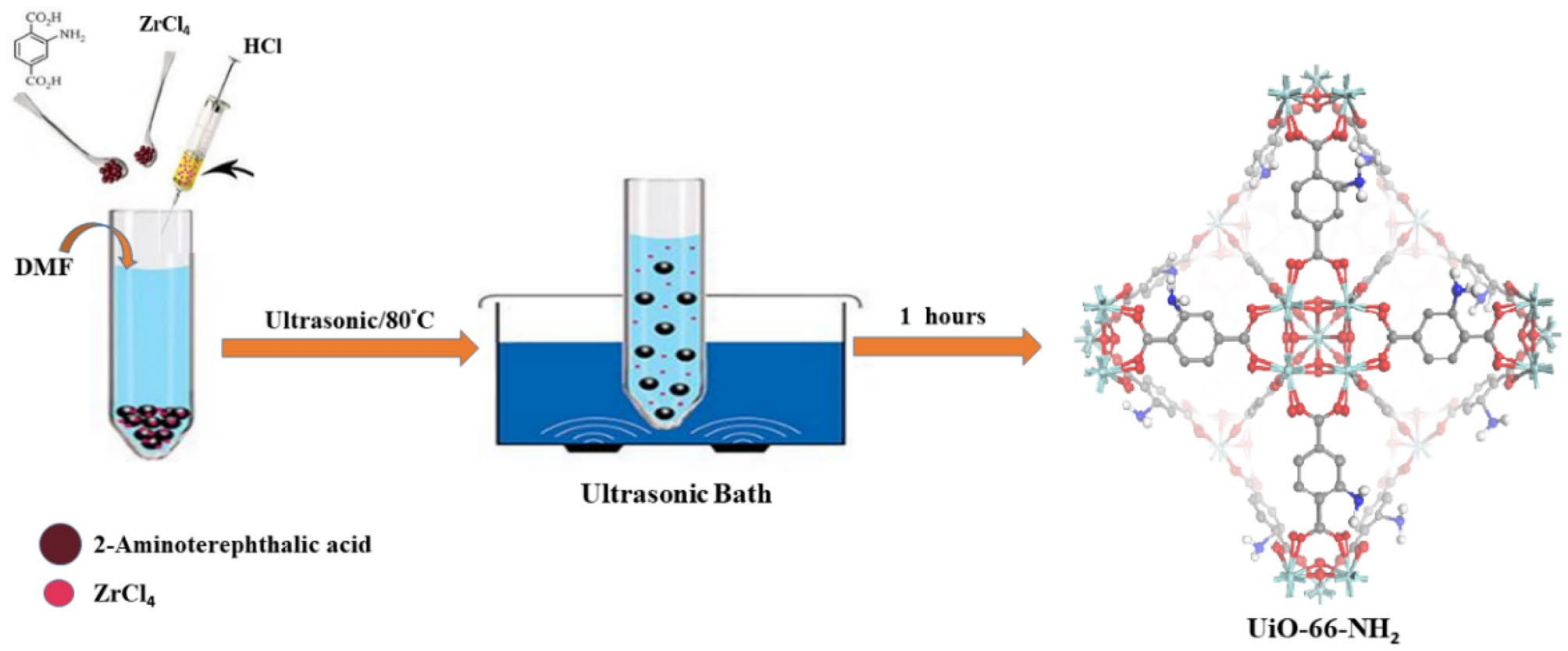
The Preparation of UiO-66-NH_2_ by the sonochemical method.

The sonochemical synthesis of the UiO-66-NH_2_ is such that the first 125.84 mg (0.54 mmol) of ZrCl_4_ was added to 5 mL of DMF, and then 1 mL of concentrated hydrochloric acid was surcharged into the hybrid system to become clear under ultrasound waves. Then 135.86 mg (0.75 mmol) of 2-amino terephthalic acid was dissolved in 10 mL of DMF and appended to the prepared ZrCl_4_ solution. The following synthesis of the UiO-66-NH_2_ was carried out with ultrasonic equipment at 80 °C and atmospheric pressure for 60 min with fixed concentrations of chemical materials (Scheme 1). After centrifuging at 8000 rpm for 10 min, sediments were collected and washed three times with fresh DMF. By immersing the samples in MeOH, the DMF molecules trapped within the pores of the framework were removed by solvent exchange. The MeOH exchange process was repeated three times over three days with the solvent being refreshed every 24 h. Thereafter, UiO-66-NH_2_ was activated overnight under a static vacuum (50–100 mTorr) at a temperature of 100 °C.

### Experimental equipment

The adsorption isotherm is measured by releasing pure gases, such as CO_2_ or N_2_, from a gas storage cylinder (purity level of 99.99%). Subsequently, these gases undergo a heating process facilitated by their passage through an electric heater. Upon exiting the heater, the gases are introduced into a mixing tank, with temperature and pressure being uniform within the tank. The processed gas is then conveyed to the reactor, where it interfaces with the adsorbent material. To monitor and regulate the operational parameters, the reactor incorporates pressure and temperature sensors. The sensors facilitate the continuous measurement of gas pressure and temperature, with the acquired data being transmitted to a controlling mechanism. Through the manipulation of the heating output, the controller effectively sustains the reactor temperature at a predetermined set point. Simultaneously, a computer device logs the pressure and temperature data on a per-second basis, ensuring a comprehensive record of the process. The equations detailing the computational framework utilized for the quantification of adsorption capacity are specified as Eq. (S1–S5)^59^.

The precision of the fitted models concerning the empirical data was assessed through the utilization of the correlation coefficient error (R^2^). This metric signifies the proportion of variations observed in the reliant variable, specifically the variance within the mean. The presented equation is expressed in the subsequent manner^60^:

1 $${R}^{2}=\frac{{({q}_{{e}_{measured}}-\overline{{q }_{{e}_{calculated}}})}^{2}}{\sum {({q}_{{e}_{measured}}-\overline{{q }_{{e}_{calculated}}})}^{2}+\sum {({q}_{{e}_{measured}}-{q}_{{e}_{calculated}})}^{2}}$$

## Results and discussion

### Adsorbent characterization

**Figure 1:**
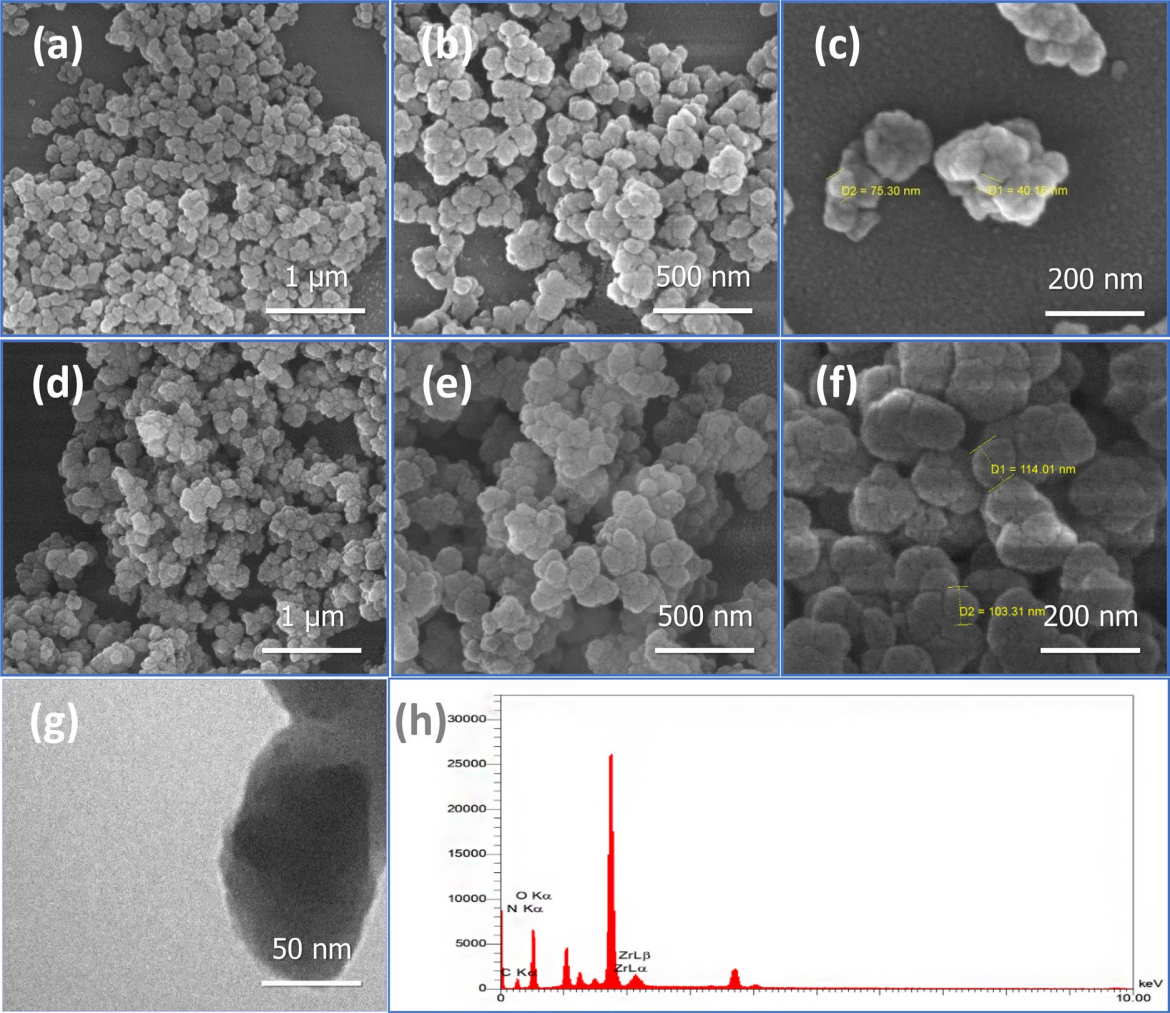
SEM micrograph of (**a–c**) UiO-66-NH_2_ synthesized by sonochemical method, and (**d–f**) UiO-66-NH_2_ (solvothermal) in different sizes. TEM (**g**) and EDX (**h**) of UiO-66-NH_2_ synthesized by sonochemical method.

FESEM was used to investigate the morphology and particle size of UiO-66-NH_2_ synthesized by the sonochemical method. The FESEM images in Fig. 1a–f show regularly shaped particles with a diameter of less than 100 nm, which is much smaller than the diameter of UiO-66-NH_2_ synthesized via solvothermal strategy. The UiO-66-NH_2_ synthesized by the sonochemical method exhibits symmetrical crystals with a sphere-shaped morphology, while UiO-66-NH_2_ also showed the same shape, but with larger crystals. Through the use of TEM, the exact size and morphology of the synthesized UiO-66-NH_2_ nanoparticles were determined. As shown in Fig. 1g, UiO-66-NH_2_ formed dense spherical crystals with an average size of less than 100 nm. Also, the EDX analysis of compounds showed peaks related to the Zr, C, N, and O elements Fig. 1h. Both MOFs synthesized in this study have similar crystal morphologies and EDX to those described in the literature^61^.

**Figure 2:**
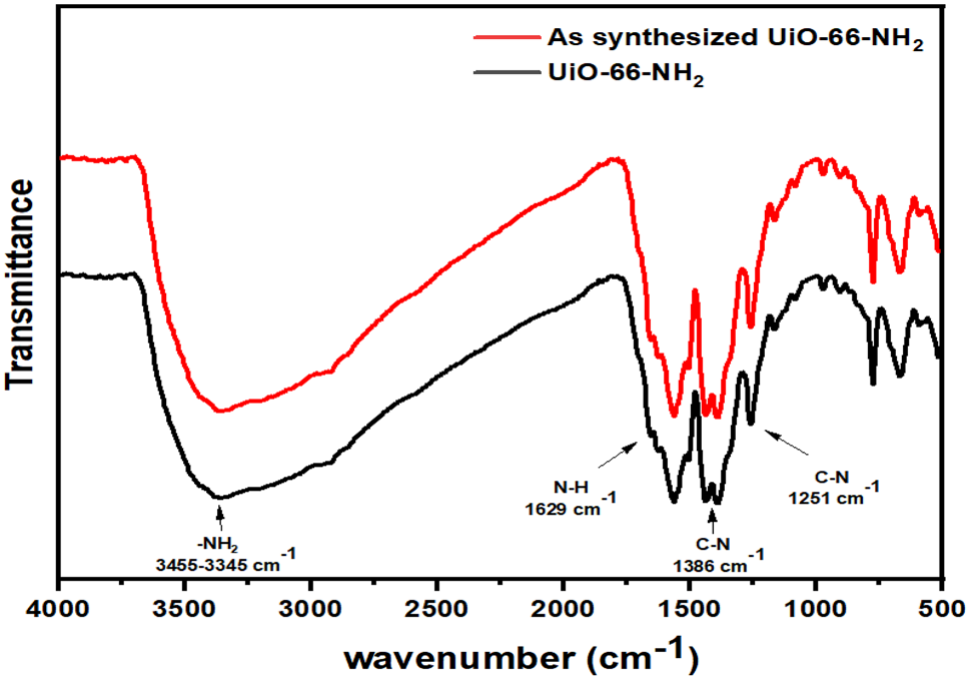
FT-IR spectra of the UiO-66-NH_2_ simulated by solvothermal method, and the as-synthesized UiO-66-NH_2_ by sonochemical method.

FT-IR spectra of the synthesized UiO-66-NH_2_ are presented in Fig. 2. In the FT-IR spectra of UiO-66-NH_2_ simulated (black) and synthesized by sonochemical (red) methods, the bands associated with the asymmetric and symmetric stretching vibrations of carboxylate group (COO^−^), in the aromatic amine group, N–H bending vibrations and C–N stretching vibrations were observed at 1568, 1629, 1386, and 1251 cm^−1^, respectively, which are consistent with those reported in the type of literature examined^61^.

**Figure 3:**
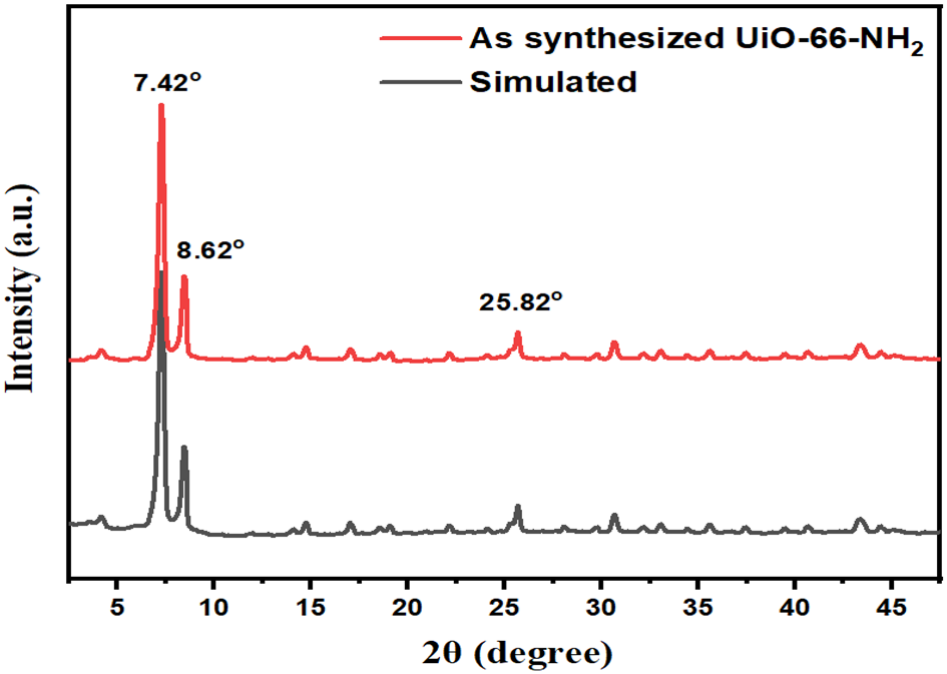
PXRD patterns of the UiO-66-NH_2_ simulated (CCDC No. 889529)^34^, and the as-synthesized UiO-66-NH_2_ by sonochemical method.

The PXRD patterns of UiO-66-NH_2_ simulated, and the as-synthesized UiO-66-NH_2_ by sonochemical method, are given in Fig. 3. The characteristic peaks of the as-synthesized UiO-66-NH_2_, which are shown in the figure matched well with the XRD patterns previously reported (CCDC No. 889529)^61,62^. Sharp peaks in the XRD patterns of the sample indicate a high degree of crystallinity. The results indicate that as-synthesized UiO-66-NH_2_ by the sonochemical method was well prepared, and its structure is impurity-free.

**Figure 4:**
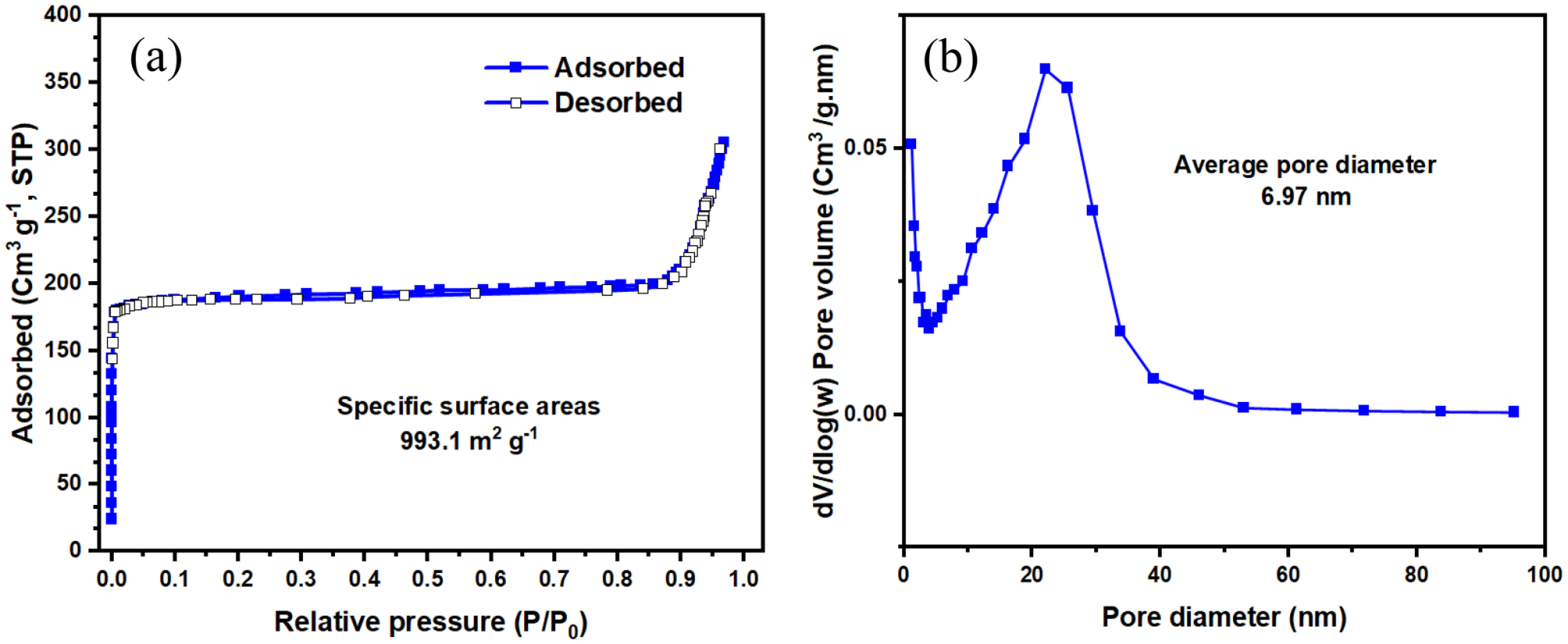
(**a**) BET (surface area) and (**b**) BJH (pore diameter) of UiO-66-NH_2_ synthesized by the sonochemical method.

The surface area (BET method) and pore diameter (BJH method) of UiO-66-NH_2_ synthesized by the sonochemical method are shown in Fig. 4. It can be observed that the structures of each sample featured the type I isotherm which has a hysteresis that indicates the presence of micropores in the material^63^. The BET surface area of UiO-66-NH_2_ synthesized by the solvothermal method was 876 m^2^/g with a pore volume of 0.38 cm^3^/g^64^ while the BET surface area of the UiO-66-NH_2_ synthesized by the sonochemical method was obtained at 993.1 m^2^/g with a pore volume of 0.14 cm^3^/g. The results showed that in the sonochemical synthesis method, not only was it synthesized in a short time (1 h) and with the easier UiO-66-NH_2_ method, but the surface area showed a significant increase. This advantage is very important in MOF synthesis because it optimizes the surface area. Also, the larger the surface area of the sample, the better gas adsorption occurs.

**Figure 5:**
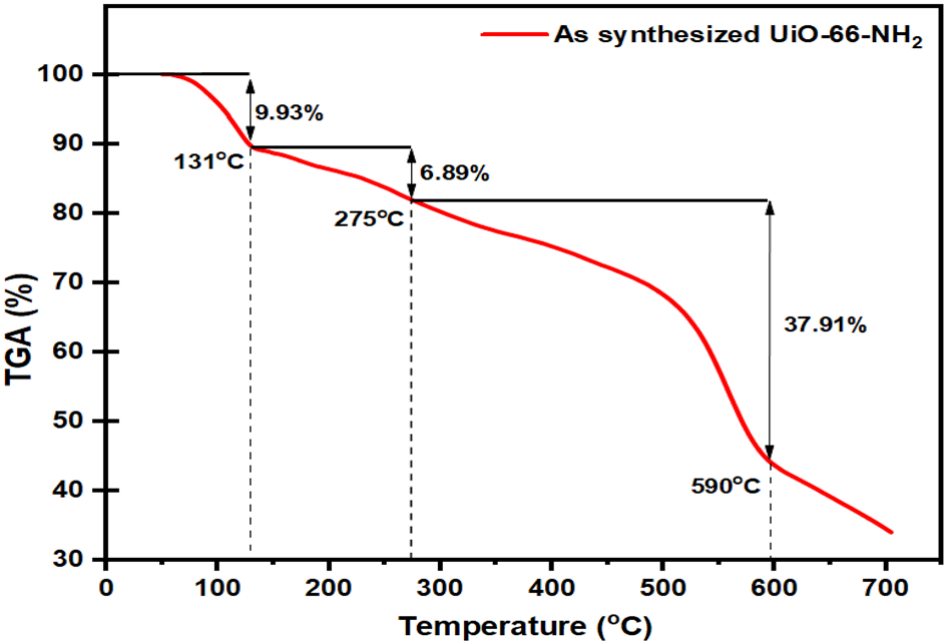
TGA curve of synthesized UiO-66-NH_2_ by sonochemical method.

As shown in Fig. 5, TGA measurements were conducted in an air atmosphere from 40 to 750 °C to determine the thermal stability of UIO-66-NH_2_. The sample loses approximately 9.93% of its weight at 20–131 °C due to moisture. It has been found that weight loss is approximately 6.89 and 42.43% when the temperature is raised further, up to 275 °C and 590 °C, respectively.

UiO-66-NH_2_ has a chemical formula of ZrO(H_2_O)_1/3_C_8_H_3_NH_2_O_4_, according to the literature^65^.

2 $$ZrO{(H}_{2}{O)}_{1/3}{C}_{8}{H}_{3}{NH}_{2}{O}_{4}\left(wet\right)\longrightarrow^{\!\!\!\!\!\!\!\!\!\!\!\!\!\!\!lost \, 9.83{\% }} ZrO{(H}_{2}{O)}_{1/3}{C}_{8}{H}_{3}{NH}_{2}{O}_{4}\left(dry\right) \longrightarrow^{\!\!\!\!\!\!\!\!\!\!\!\!\!\!\!lost \,6.89{\% }} ZrO{C}_{8}{H}_{3}{NH}_{2}{O}_{4}(S) \longrightarrow^{\!\!\!\!\!\!\!\!\!\!\!\!\!\!\!lost\, 37.91{\% }}  ZrO({OH)}_{4}(S)$$

The TGA results approximate the theoretical weight loss rate (%) of UiO-66-NH_2_ thermal decomposition according to Eq. (2).

### Assessment of CO_2_/N_2_ adsorption

#### Experimental gas adsorption measurements

Adsorption isotherm represents a crucial curve that explains the process governing how a substance moves from porous materials or fluid environments to a solid surface at a constant temperature, either being held onto or released^66,67^. Adsorption equilibrium occurs when the amount of substance attached to the solid reaches a balance with the amount remaining in the solution. This equilibrium is achieved when the substance-containing phase has been in contact with the solid material for a sufficient time, resulting in a dynamic balance between the substance's concentration in the bulk solution and at the solid–fluid interface^68,69^. In this section, the adsorption capacity efficacy of UiO-66-NH_2_ MOF, fabricated using an innovative sonochemical procedure, is compared with that of the conventional solvothermal method through empirical analysis. The assessment encompasses two distinct adsorbents, conducted at a temperature of 25 °C, 50 °C, and varying pressures, to elucidate their respective isotherm characteristics. In this context, two separate pressure ranges were employed to evaluate the capacity of the adsorbents under both low and high-pressure conditions.

**Figure 6:**
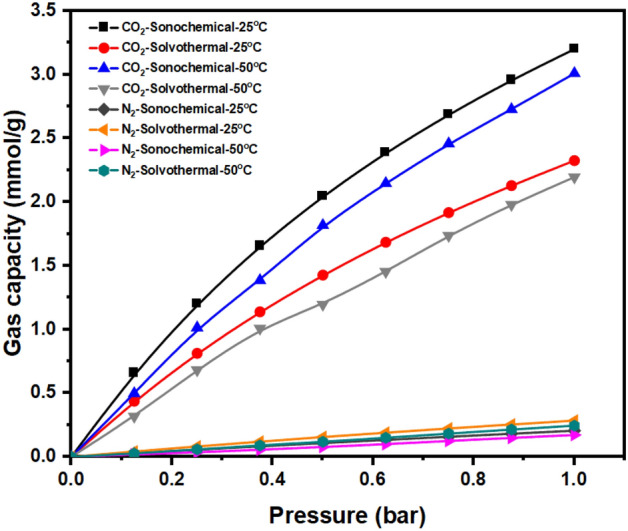
Gas adsorption experimental isotherm of sonochemical and solvothermal UiO-66-NH_2_ at 25 °C, 50 °C, and low pressures.

The adsorption capacity for CO_2_ of UiO-66-NH_2_ MOF, synthesized utilizing the sonochemical method, demonstrates a noteworthy enhancement in comparison to the conventionally synthesized UiO-66-NH_2_ MOF through the solvothermal approach. As illustrated in Fig. 6, the conventional UiO-66-NH_2_ exhibits a CO_2_ capacity of 2.32 mmol/g, while the sonochemically derived UiO-66-NH_2_ showcases an adsorption capacity of 3.2 mmol/g at 1 bar and 25 °C. This augmentation can be attributed to the higher surface area and smaller particle sizes inherent in the latter material^70^. The amplified surface area offers a greater number of adsorption sites available for gas molecules to interact with the surface. Consequently, this results in an elevated adsorption capacity, even under conditions of low pressure. Under elevated pressures, owing to the greater surface area afforded by the sonochemically synthesized UiO-66-NH_2_, it was foreseeable that this particular adsorbent would demonstrate an augmented capacity for CO_2_ gas molecules, as discernible from the findings presented in Fig. S1. The conventional UiO-66-NH_2_ registers a CO_2_ adsorption capacity of 14.72 mmol/g, whereas the sonochemical counterpart, UiO-66-NH_2_, showcases a capacity of 18.05 mmol/g under conditions of 20 bar and 25 °C. The noteworthy improvement in CO_2_ adsorption capacity demonstrated here enhances the promise of the novel sonochemical method employed in this study.

#### Isotherm modeling

Isotherm relationship is expressed mathematically, which plays a significant role in modeling, analyzing, designing, and practicing adsorption systems. This relationship is commonly visualized by graphing the amount of substance on the solid surface against the remaining concentration in the solution^71^. The physical and chemical properties of the system, along with the underlying thermodynamic assumptions, offer insights into the mechanism of adsorption, the characteristics of the surface, and how strongly the solid material attracts the substance^72^.

The Langmuir adsorption isotherm was initially formulated to elucidate gas-to-solid phase adsorption phenomena; subsequently, its applicability extended to encompass solid–liquid interfaces. The degree of surface coverage is expressed in terms of fractional coverage and is contingent upon the concentration of the adsorbate. The mathematical derivation is predicated upon the inherent conceptual simplicity of the underlying mechanisms, in conjunction with several presumptions: (1) uniform homogeneity across the surface, indicating energetically homogeneous sites; (2) adsorption transpires as a monomolecular layer process, with each site exclusively accommodating a solitary adsorbate molecule; (3) absence of lateral interactions among adsorbed molecules; and (4) the reversibility of the adsorption process^73^ (Eq. 3).

3 $${q}_{e}=\frac{{q}_{m}{K}_{L}{P}_{e}}{1+{K}_{L}{P}_{e}}$$

The Freundlich isotherm possesses the capacity to delineate non-ideal, multilayer, and reversible adsorption transpiring at a heterogeneous surface. This isotherm additionally postulates the existence of diverse binding energies among the adsorption sites. The energy distribution encompassing adsorptive sites, as delineated within the framework of the Freundlich isotherm, manifests as a spectrum of varying binding energies, rather than a uniform energy distribution. This distribution adheres to an exponential-type function, closely approximating the complexities of actual adsorption scenarios^74^ (Eq. 4)*.*

4 $${q}_{e}={K}_{F}{P}^\frac{1}{n}$$

The Dubinin–Radushkevich isotherm represents an empirical framework originally devised to elucidate the adsorption behavior of subcritical vapors onto micropore solids through a pore-filling mechanism. Typically, it finds application in characterizing adsorption processes involving a Gaussian energy distribution on heterogeneous surfaces^75^ (Eqs. 5, 6).

5 $${q}_{e}={q}_{m}\,exp(-{\beta \omega }^{2})$$

6 $$E=\frac{1}{{\left(2\beta \right)}^{0.5}}$$

The variables q_e_ and q_m_ denote the equilibrium quantity and the maximum capacity of adsorption for CO_2_ and N_2_ gases (expressed in mmol/g). The parameter P_e_ represents the pressure prevailing during the equilibrium state (measured in bar). The Freundlich model is characterized by the constants K_F_ (mmol/g.bar^1/n) and n, while the Langmuir model is defined by the constant K_L_. Within the Dubinin-Radushkevich (D-R) model, the symbol β signifies a model-specific constant (quantified in mol^2^/J), while the term ω pertains to the Polanyi potential (measured in J/mol) and E (kJ/mol) represents the average energy of sorption when it moves from the bulk solution to the solid surface^76^. The graphical depiction of the pertinent fitting models can be observed in Fig. S2, within the context of a stable temperature environment set at 25 °C.

Fitting values of isotherm models with their respective R^2^ values at 25 °C are reported in Table 1. Drawing from an assessment of the average coefficients of determination (R^2^) obtained from diverse isotherm models, it becomes evident that the Langmuir model exhibits a higher degree of precision when compared to alternative models. This alignment with existing scholarly literature is noteworthy, as the preponderance of Metal–Organic Frameworks (MOFs) predominantly rely upon the Langmuir isotherm model to illuminate their behavioral characteristics^77^. Based on the content expounded within this section, it can be compellingly inferred that the phenomenon of monolayer adsorption onto the surface of MOFs is indeed transpiring. Furthermore, a notable consequence of this study's findings pertains to the discernment of an absence of lateral interactions among the adsorbed species. Moreover, it is intimated that the suitability to reversibility inherent in this adsorption process surmounts the comparably intricate dynamics intrinsic to alternative adsorption mechanisms^78^.

**Table 1:** Isotherm models parameters for CO_2_ and N_2_ adsorption at 25 °C.

Isotherm model	Parameters	CO_2_ adsorption	N_2_ adsorption
Langmuir	q_m_	7.222	1.702
	K_L_	0.793	0.200
	R^2^	0.999	0.999
Freundlich	K_F_	3.262	0.286
	n	1.413	1.115
	R^2^	0.998	0.996
Dubinin–Radushkevich	q_m_	3.778	0.363
	β	0.074	0.104
	E	2.595	2.190
	R^2^	0.989	0.988

#### Adsorption kinetic evaluation

The rate of adsorption is additionally regulated by adsorption kinetics, playing a pivotal role in dictating the temporal extent essential for achieving adsorption equilibrium. Kinetic models offer insights into the trajectories of adsorption and the potential mechanisms at play. This data holds significance in the advancement of the process and the design of the adsorption system^79^.

The pseudo-first-order model operates under the premise that the rate of change of solute adsorption over time is directly correlated to the disparity between saturation concentrations and the quantity of solid adsorption as time progresses. This principle is generally valid during the initial phases of an adsorption procedure (Eq. (7))^80^.

7 $${q}_{t}={q}_{e}(1-{e}^{{k}_{1}t})$$

The theoretical framework of the pseudo-second-order kinetic model rests upon the supposition that the governing factor in the rate of adsorption is the process of chemical sorption or chemisorption. This model offers projections encompassing the entirety of adsorption dynamics. In this scenario, the pace of adsorption is determined by the capacity for adsorption, rather than being influenced by the concentration of the adsorbate (Eq. 8)^81^.

8 $${q}_{t}=({q}_{e}^{2} {k}_{2}t)/( 1+{q}_{e}{k}_{2}t)$$

The elovich kinetic model finds frequent application in the interpretation of adsorption kinetics and effectively characterizes second-order kinetics, under the premise that the surface exhibits energetic heterogeneity (Eq. 9)^82^.

9 $${q}_{t}=\frac{1}{\beta }\mathit{ln}\left(\alpha \beta \right)+ \frac{1}{\beta } ln(t)$$

Here, q_t_, K_1_, and K_2_ denote the adsorption capacity, the rate constant for the pseudo-first-order model, and the rate constant for the pseudo-second-order model, respectively. Within the framework of the Elovich model, the parameter "α" signifies the initial rate of adsorption (mg/g.min), while the parameter "β" represents the constant associated with the desorption process (g/mg).

**Figure 7:**
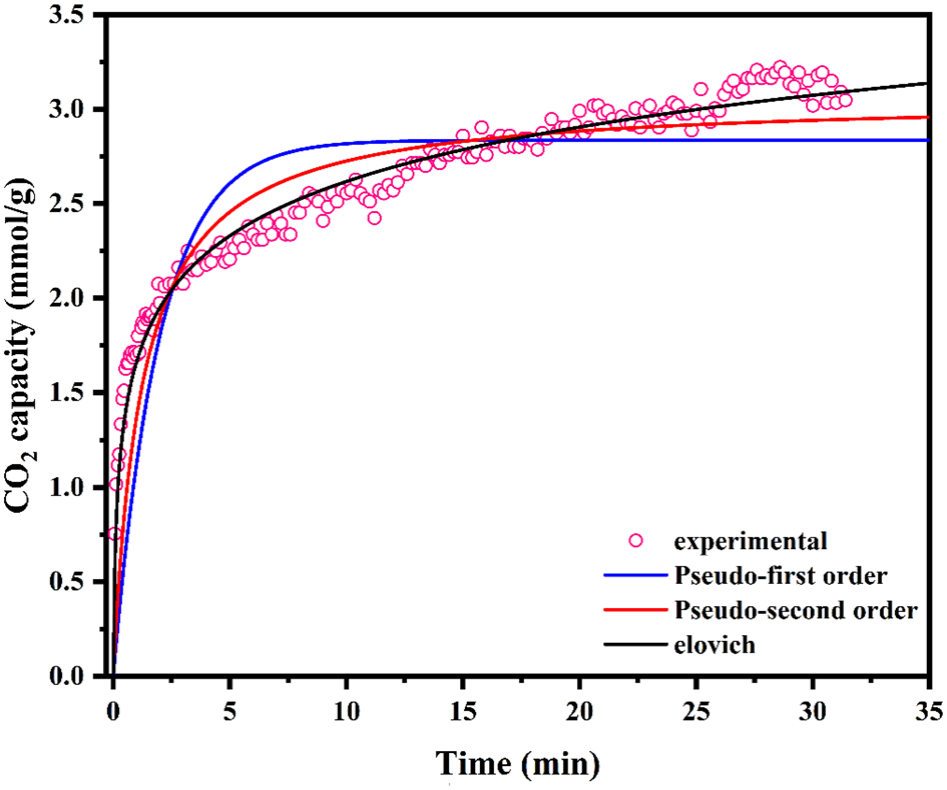
Assessing kinetic models and CO_2_ adsorption experimental data for sonochemical UiO-66-NH_2_ under conditions of 25 °C and 1 bar.

Based on the observations presented in Table 2 and the correlation coefficient (R^2^) values associated with the kinetic models, the Elovich model may be selected as the most suitable model. Drawing from the parameters derived via the Elovich kinetic model, it becomes evident that the initial adsorption rate stands impressively at 22.72 mg/g∙min. This particular value carries significance within the CO_2_ capture domain due to its substantial nature. Simultaneously, the desorption constant, fixed at 2.408 g/mg, emerges as notably high. This elevated desorption constant introduces a challenge when it comes to facilitating the desorption process—a less favorable aspect for integrating energy in industrial processes. Higher desorption constants contribute to a more complicated desorption phase, making it tougher to release adsorbed molecules smoothly. This poses a hitch in terms of energy efficiency and overall integration of industrial operations. On the other side, lower values of this parameter indicate that the adsorbent material isn't very inclined to hold onto adsorbate molecules. This might seem advantageous for facilitating desorption, but it could also affect the effectiveness of the adsorption process itself. The kinetic models employed in this study and their corresponding fittings are illustrated in Fig. 7.

**Table 2:** Kinetic model parameters for CO_2_ adsorption at 25 °C.

Kinetic model	Parameters	CO_2_ adsorption
Pseudo-first order	q_e_	2.836
	K_1_	0.504
	R^2^	0.822
Pseudo-second order	q_e_	3.062
	K_2_	0.265
	R^2^	0.915
Elovich	α	22.72
	β	2.408
	R^2^	0.981

#### Isosteric heat of adsorption

In the realm of thermodynamic attributes, a preeminent parameter employed to assess the extent of interaction between CO_2_ molecules and the surfaces of adsorbents pertains to the isosteric heat of adsorption. This parameter plays a pivotal role in the formulation and advancement of gas separation methodologies of notable industrial significance, such as pressure-swing adsorption (PSA) and temperature-swing adsorption (TSA)^83^. This bears substantial significance in the realm of industrial gas separation processes. The significance of the isosteric heat of adsorption (Q_st_) magnitude lies in its ability to delineate the interplay between the adsorbent and the adsorbate species. This metric serves as a descriptor for the robustness of the adsorption phenomenon, with an escalated Q_st_ denoting a more pronounced interaction between the adsorbed substance and the adsorbing material. Notably, an elevated Q_st_ value corresponds to intensified bonding between the adsorbate and the adsorbent, albeit accompanied by a concomitant escalation in regeneration expenses. The quantification of the isosteric heat of adsorption finds its mathematical expression in the Clausius–Clapeyron equation as follows^84^:

10 $${Q}_{st}=-R{\left[\frac{\partial \left(lnP\right)}{\partial \left(\frac{1}{T}\right)}\right]}_{n}$$

**Figure 8:**
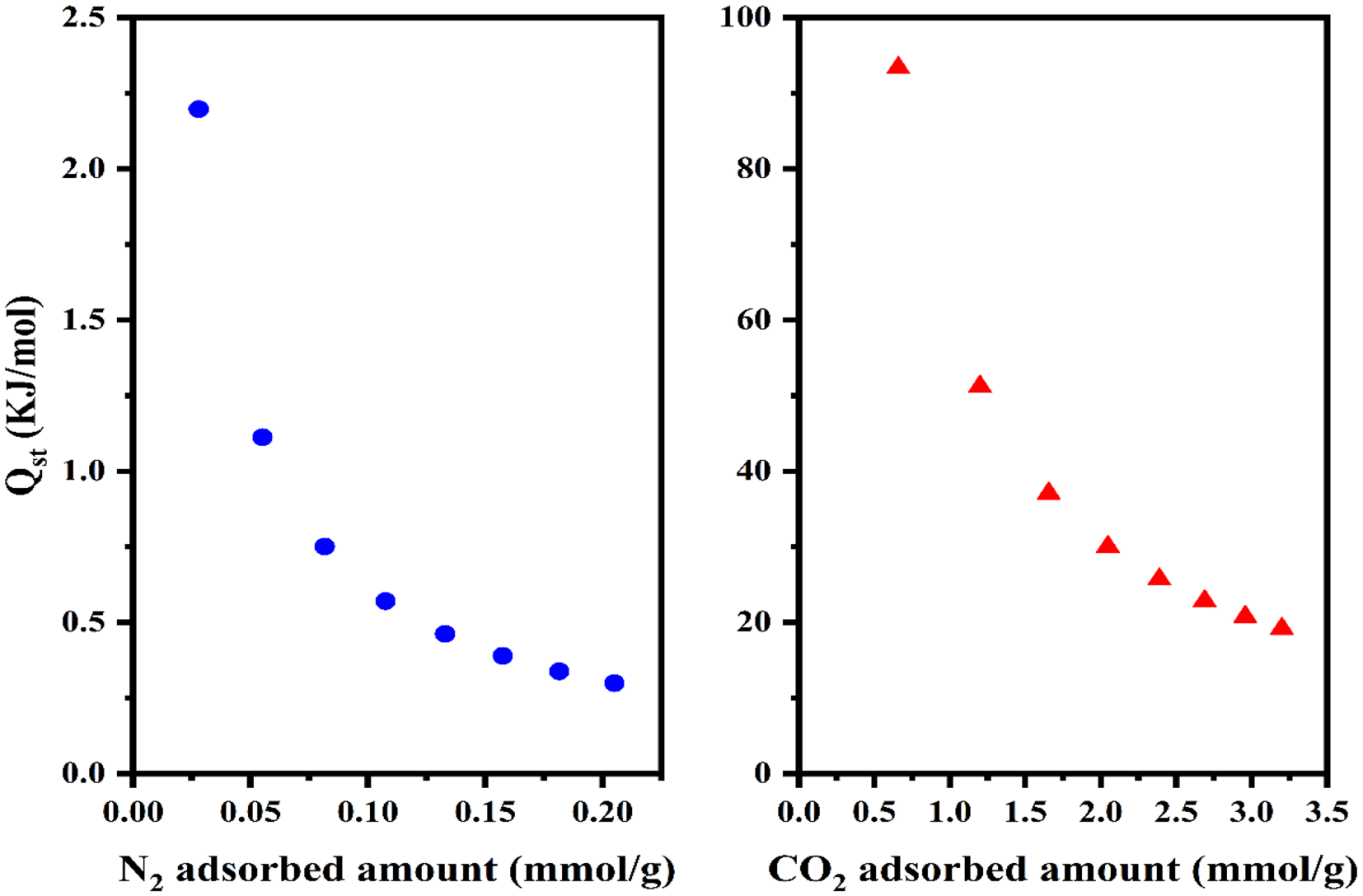
Isosteric heat of adsorption for sonochemical UiO-66-NH_2_.

Herein, Q_st_ represents the isosteric heat of adsorption at a constant level of adsorbed quantity denoted as n [mmol/g], while R stands for the universal gas constant [J/mol⋅K]. As depicted in Fig. 8, the initial heat of adsorption registers a value exceeding 80 kJ/mol, serving as an indicative marker of chemisorption events transpiring at the surface of the Metal–Organic Framework (MOF)^85^. This notable energy association between the adsorbate and the adsorbent comes from the presence of amine functional groups inherent within the structural framework of UiO-66-NH_2_. As the dynamic process of adsorption advances at the interface, the isosteric heat experiences a proportionate decline, primarily attributed to the progressive occupation of adsorption sites and the concomitant reduction in available surface domains for the adhesion of adsorbate entities. This phenomenon is intricately intertwined with the evolving interplay between the adsorbent surface and the adsorbate molecules. Additionally, the low isosteric heat value for nitrogen adsorption aligns with the low adsorption amount observed. This could be due to nitrogen molecules having weak electrostatic interactions with the adsorbent surface.

#### IAST-based selectivity

Upon its initial inception by Myers and Prausnitz^86^, the IAST methodology was conceived. This theoretical framework mandates the availability of distinct single-component isotherms corresponding to each gas species. This prerequisite facilitates the prognostication of both selectivity and adsorption behaviors for every constituent present within a mixture comprising the aforementioned gas species. In scenarios featuring a binary mixture, a set of four equations (Eq. S6–S9) necessitates resolution in order to derive values for ${P}_{B}^{*}$ and ${P}_{C}^{*}$. The successful resolution of this equation set is imperative for attaining a comprehensive grasp of the compositional attributes characterizing the system^87^**.**

**Figure 9:**
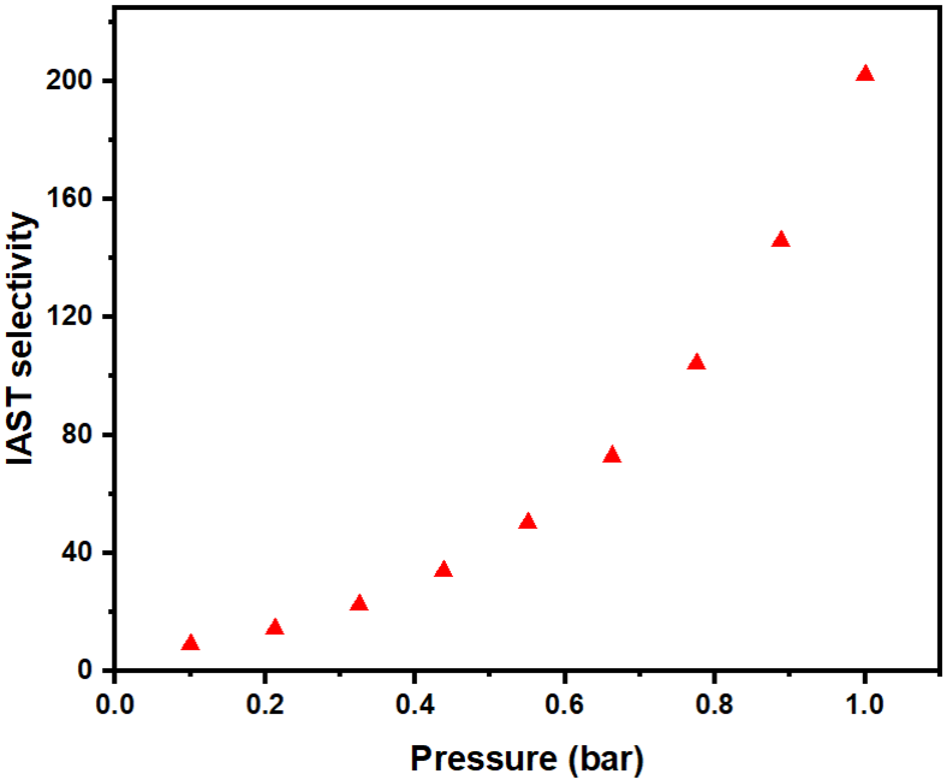
IAST-based selectivity of sonochemical UiO-66-NH_2_ at 25 °C and 15% CO_2_ and 85% N_2_.

In the present section, an assessment of selectivity was conducted through the utilization of Ideal Adsorbed Solution Theory (IAST) calculations. These calculations were performed under the representative gas composition of flue gases, comprised of 15% CO_2_ and 85% N_2_^88^ As delineated in Fig. 9, the sonochemical variant of UiO-66-NH_2_ demonstrates a notable preference for CO_2_, particularly observable at an operating pressure of 1 bar. The discernible and quantifiable escalation in the degree of selectivity, conspicuously coincident with a progressively augmented operational pressure, can be expounded upon in the context of an expanded reservoir of CO_2_ molecules, thereby affording an increased propensity for intricate and selective interactions with the functional amine groups. This intriguing phenomenon, which inherently governs the distinctive predilection for CO_2_ adsorption, finds its theoretical foundation in the underlying non-reactive attributes inherent to N_2_ molecules, compelling them to exhibit limited affinity for engagement with the said amine groups^89^, The selective molecular dynamics underscored by this intriguing behavior can be further rationalized by invoking the broader intermolecular forces at play. Specifically, the notable exclusion of N_2_ molecules from active participation in adsorptive processes can be attributed to the distinct electronic and steric attributes inherent to these molecules, rendering them less inclined to form robust and chemically meaningful interactions with the amine functionalities. This, in turn, perpetuates the prevailing selectivity bias towards CO_2_. In this intricate molecular interplay, the intricate framework of attractive and repulsive forces governing the interactions between nitrogen species comes into play. The intrinsic repulsive forces operative between nitrogen molecules provide an additional facet to the observed selectivity enhancement. The repulsion that manifests when nitrogen molecules approach each other discourages their aggregation, further segregating them from the more favored CO_2_ molecules that are amenable to productive interaction with the functional groups^90^.

#### Adsorption mechanism

**Scheme 2:**
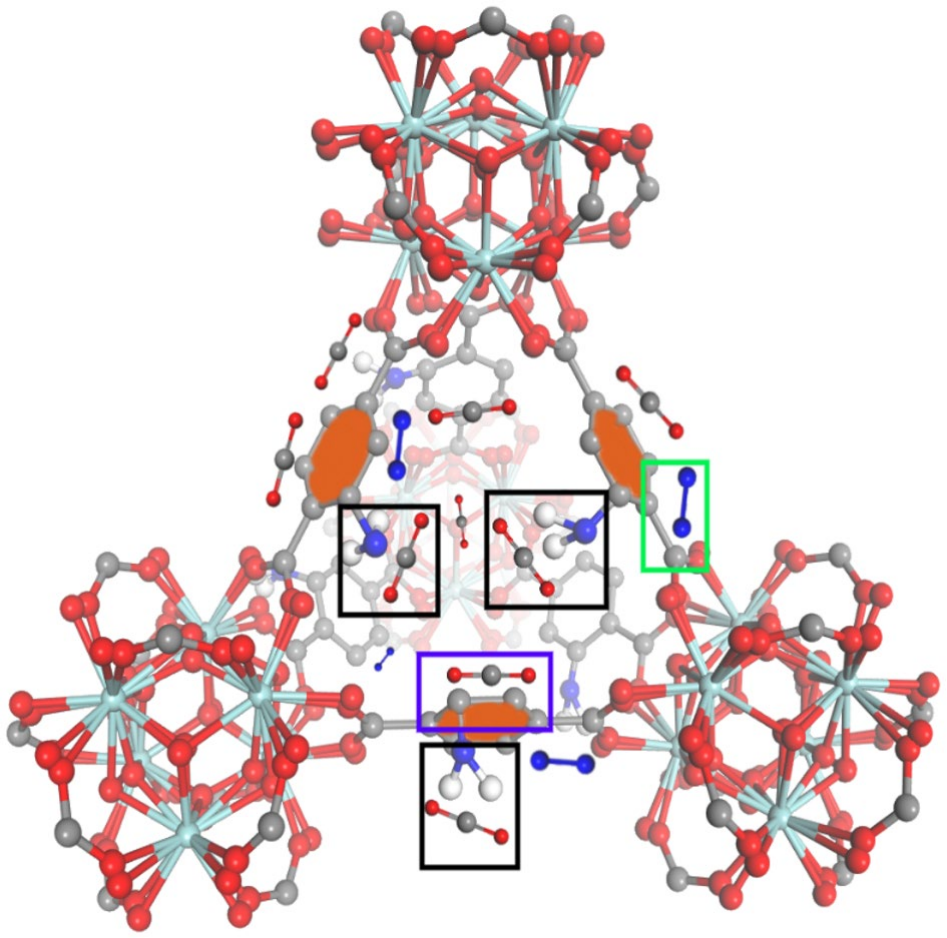
CO_2_ and N_2_ molecules adsorption mechanism.

The principal mechanism governing the adsorption of CO_2_ and N_2_ molecules onto UiO-66-NH_2_ involves their chemisorption onto the amine functional groups located on the surface. In the context of this chemical reaction, the amine species (RNH_2_) undergoes a reaction with carbon dioxide (CO_2_), resulting in the generation of an amine carbamate substance (RNHCOO^−^)^91^. In the context of physisorption, benzene rings exhibit resonance structures that produce a state of delocalized electron density within the ring structure^92^. This phenomenon leads to the accumulation of heightened negative charges at the center of the benzene rings, thereby giving rise to an elevated field gradient. In our scenario, where both adsorbates possess different quadrupole moments, the comprehensive interaction between a homogeneous electric field and the quadrupole yielded zero. Nevertheless, a significant interaction proceeds between the quadrupole and the gradient of the electric field. Consequently, the electrostatic interaction provokes the adsorption of CO_2_ molecules by the benzene rings, with certain N_2_ molecules adsorbed onto the peripheral regions of these benzene rings. All this information is visually depicted in Scheme 2, wherein the black boxes exemplify the phenomenon of chemisorption, the blue box signifies the electrostatic interaction of CO_2_ molecules situated at the center of benzene rings, and the green box illustrates the adsorption of N_2_ molecules on the outer peripheries of the benzene rings.

#### Cyclic stability of adsorbent

The assessment of an adsorbent's stability and regenerability holds significant importance in the evaluation of its appropriateness for CO_2_ capture procedures^1^. In order to gauge the reusability of sonochemical UiO-66-NH_2_, a sequence comprising 8 adsorption cycles was executed under conditions of 25 °C and 1 bar. After this, the adsorbents underwent recycling via exposure to a vacuum oven at a temperature of 150 °C for a duration of 8 h. The findings, depicted in Fig. S3, underscore the remarkable stability exhibited by sonochemical UiO-66-NH_2_ throughout the span of the eight cycles compared to solvothermal UiO-66-NH_2_. The cyclic examinations reveal a negligible reduction in CO_2_ capacity for the adsorbents, indicating a mere 0.6 mmol/g decline for sonochemical UiO-66-NH_2_ and 0.58 mmol/g decrease for solvothermal UiO-66-NH_2_. This great stability can be ascribed to the presence of amine groups that are grafted in the structure, in conjunction with the small particle dimensions. This observation underscores the prowess of sonochemical UiO-66-NH_2_ as a highly promising contender for the CO_2_ capture process, given its capacity to sustain a noteworthy level of CO_2_ adsorption even after undergoing 8 consecutive cycles. In sum, the exceptional stability and regenerability demonstrated by the adsorbent, combined with its elevated CO_2_ capacity and much shorter synthesis time, establish it as a propitious choice for applications in CO_2_ capture.

## Conclusions

Through experimental adsorption measurements, our investigation reveals a marked enhancement in CO_2_ adsorption exhibited by sonochemical UiO-66-NH_2_, in comparison to its solvothermal UiO-66-NH_2_ counterpart. This augmentation can be attributed to the increased surface area and smaller particle sizes characteristic of the sonochemically synthesized material. Employing isotherm modeling techniques, our analysis identifies the Langmuir isotherm as the optimal fit for the empirical data. This alignment suggests monolayer adsorption behavior, underscoring the prevalent influence of chemisorption phenomena. The application of kinetic modeling analysis yielded the Elovich model as the most appropriate fitting function, thereby emphasizing the significance of the chemisorption phenomenon. The isosteric heat analysis manifests substantial energies associated with the interaction between the adsorbent's surface and CO_2_ molecules. This observation provides further corroboration for the occurrence of chemical reactions involving the amine groups and CO_2_ gas. Utilizing the Ideal Adsorbed Solution Theory (IAST), we quantify the selectivity of the adsorbent and ascertain its notable affinity for CO_2_ under flue gas conditions, primarily attributable to the presence of amine functional groups. Additionally, an evaluation of the cyclic performance of the adsorbent underscores its viability over successive operational cycles. Specifically, the sonochemical UiO-66-NH_2_ material exhibits satisfactory stability over 8 cycles, albeit with a reduction in capacity linked to the gradual degradation of the amine groups and their interaction with CO_2_ molecules. In conclusion, the sonochemical synthesis route for UiO-66-NH_2_, characterized by augmented surface area and smaller particle sizes achievable within a concise one-hour period, produces a substantial elevation in CO_2_ adsorption capacity. Moreover, the material demonstrates pronounced selectivity for CO_2_ under flue gas conditions, ascribed to the strategic integration of amine functional groups. These findings collectively highlight the promising attributes of sonochemical UiO-66-NH_2_ as a potential candidate for advanced CO_2_ capture applications.

### Supplementary Information

Supplementary Information.

## Data Availability

The datasets used and/or analyzed during the current study available from the corresponding author on reasonable request. Adsorption capacity calculation equations, IAST calculation equations, CO_2_ adsorption capacity at high pressures, Isotherm model fitting plots, cyclic performance of adsorbent. This material is available free of charge from http://pubs.acs.org.
