# Supplementary material for: Enhanced CO2 capture potential of UiO-66-NH2 synthesized by sonochemical method: experimental findings and performance evaluation
Source: Sci Rep. 2023 Nov 14;13:19891. doi: 10.1038/s41598-023-47221-6 (PMC10645735; doi:10.1038/s41598-023-47221-6)
Supplement: Supplementary file 1 — Supplementary Information. [file 41598_2023_47221_MOESM1_ESM.docx]

**Supporting information**

Enhanced CO_2_ Capture Potential of UiO-66-NH2 Synthesized by Sonochemical Method: Experimental Findings and Performance Evaluation

*Amir Kazemi*^1^*, Fatemeh Moghadaskhou*^2^*, Mahyar Ashourzadeh Pordsari*^3^*, Faranak Manteghi*^1*^, *Azadeh Tadjarodi*^2^*,* Ahad Ghaemi^3*^

^1^ Research Laboratory of Inorganic Chemistry and Environment, Department of Chemistry, Iran University of Science and Technology, 16846-13114, Tehran, Iran

^2^ Research Laboratory of Inorganic Materials Synthesis, Department of Chemistry, Iran University of Science and Technology, 16846-13114, Tehran, Iran

^3^ School of Chemical, Petroleum and Gas Engineering, Iran University of Science and Technology, 16846-13114, Tehran, Iran

*E-mail address: f_manteghi@iust.ac.ir
 aghaemi@iust.ac.ir

1. Equations
2. Figure S1. CO_2_ adsorption isotherm of sonochemical and solvothermal UiO-66-NH_2_ at 25 °C and high pressures.
3. Figure S2. Assessing isotherm models and experimental data for sonochemical UiO-66-NH_2_ at 25 °C (a) CO_2_ adsorption (b) N_2_ adsorption.
4. Figure S3. Cyclic performance of sonochemical UiO-66-NH_2_ at 25 °C and 1 bar.

| $q=\left( \frac{VM_{W}}{\mathrm{WR}} \right)\left( \left[ \frac{P}{ZT} \right]_{f}-\left[ \frac{P}{ZT} \right]_{i} \right)$ | (S1) |
| --- | --- |
| $Z=1+\frac{\mathrm{PB}}{\mathrm{RT}}$ | (S2) |
| $B=\frac{RT_{c}}{P_{c}}(F^{0}T_{r}+\omega F^{1}T_{r})$ | (S3) |
| $F^{0}\left( T_{r} \right)=0.1446-\frac{0.330}{T_{r}}-\frac{0.1385}{T_{r}^{2}}-\frac{0.0121}{T_{r}^{3}}-\frac{0.000607}{T_{r}^{8}}$ | (S4) |
| $F^{1}\left( T_{r} \right)=0.0637+\frac{0.331}{T_{r}^{2}}-\frac{0.423}{T_{r}^{3}}-\frac{0.008}{T_{r}^{8}}$ | (S5) |
| $y_{B}=P_{B}^{*}x_{B}$ | (S6) |
| $x_{B}=\frac{P_{C}^{*}-P}{P_{C}^{*}-P_{B}^{*}}$ | (S7) |
| $\frac{1}{n_{\mathrm{tot}}}=\frac{x_{B}}{n_{B}(P_{B}^{*})}+\frac{1-x_{B}}{n_{C}(P_{C}^{*})}$ | (S8) |
| $\int_{0}^{P_{B}^{*}} \frac{n_{B}(p)}{p}dp=\int_{0}^{P_{C}^{*}} \frac{n_{C}(p)}{p}\mathrm{dp}$ | (S9) |


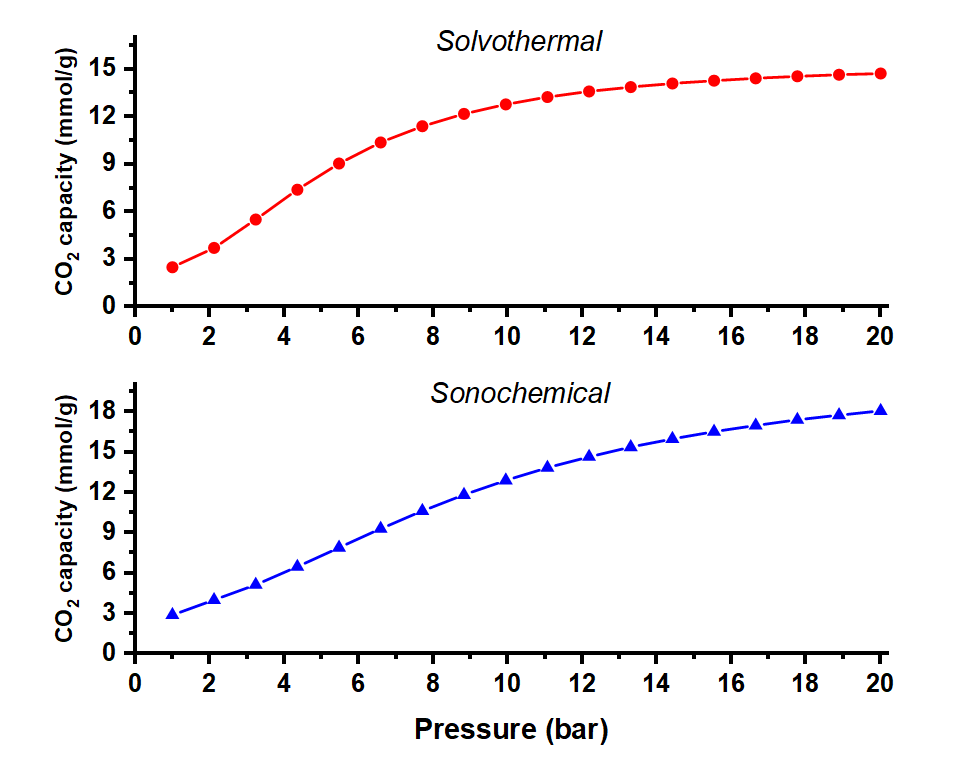


(a)

(b)

Figure S1. CO_2_ adsorption isotherm of sonochemical and solvothermal UiO-66-NH_2_ at 25 °C and high pressures.


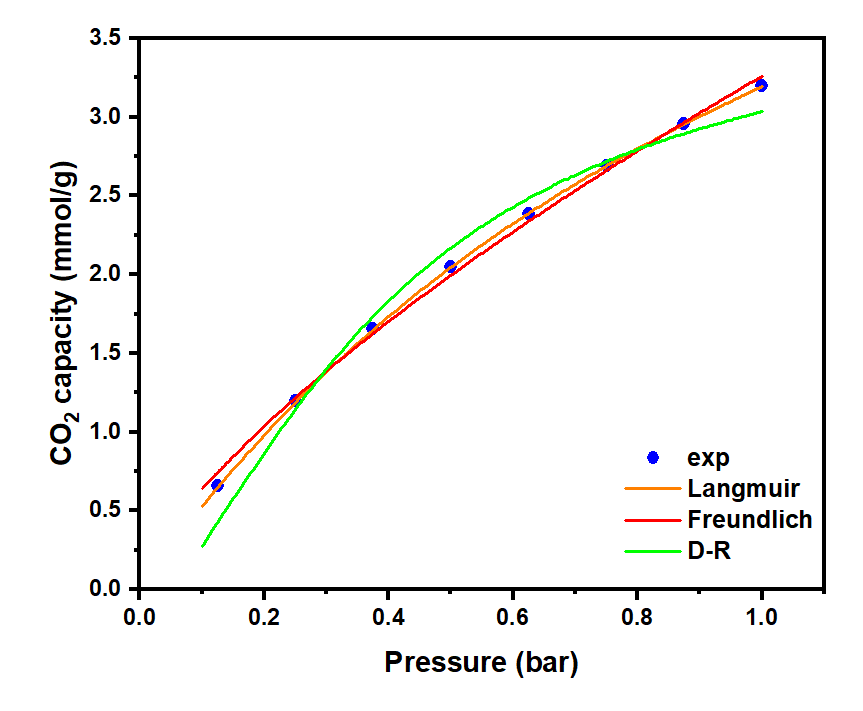

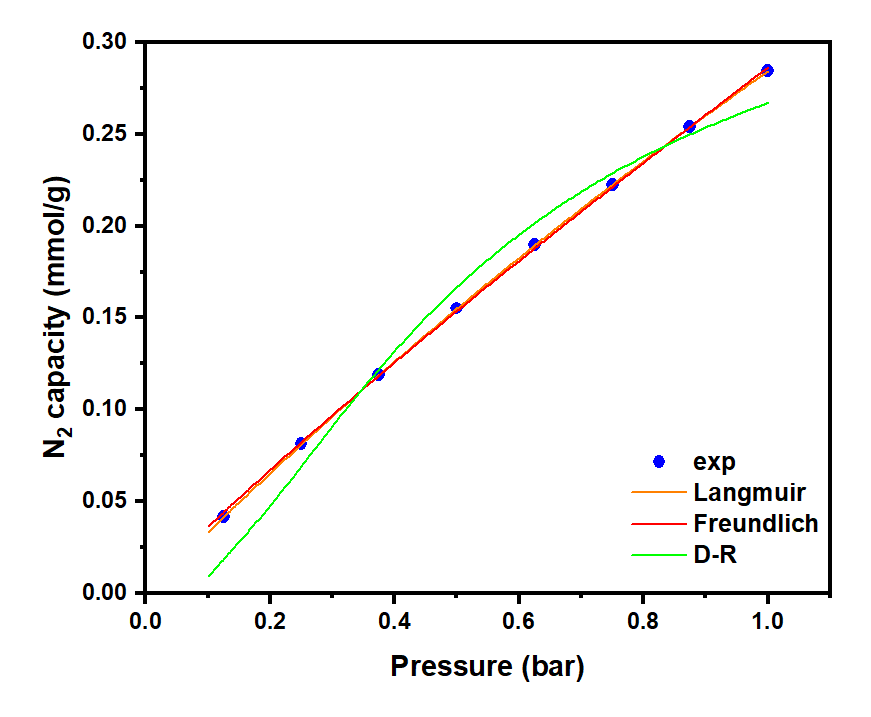


(b)

(a)

Figure S2. Assessing isotherm models and experimental data for sonochemical UiO-66-NH_2_ at 25 °C (a) CO_2_ adsorption (b) N_2_ adsorption





Figure S3. Cyclic performance of sonochemical UiO-66-NH_2_ at 25 °C and 1 bar
